# Effects of Dietary Supplementation of a Resin-Purified Aqueous-Isopropanol Olive Leaf Extract on Meat and Liver Antioxidant Parameters in Broilers

**DOI:** 10.3390/antiox12091723

**Published:** 2023-09-05

**Authors:** Konstantina Vasilopoulou, Georgios A. Papadopoulos, Styliani Lioliopoulou, Ioanna Pyrka, Nikolaos Nenadis, Soumela Savvidou, George Symeon, Vassilios Dotas, Ioannis Panitsidis, Georgios Arsenos, Ilias Giannenas

**Affiliations:** 1 Laboratory of Nutrition, Faculty of Veterinary Medicine, Aristotle University of Thessaloniki, 54124 Thessaloniki, Greece; 2 Laboratory of Animal Husbandry, Faculty of Veterinary Medicine, Aristotle University of Thessaloniki, 54124 Thessaloniki, Greece; 3 Laboratory of Food Chemistry and Technology, School of Chemistry, 54124 Thessaloniki, Greece; 4 Institute of Animal Science, Hellenic Agricultural Organisation-DEMETER, 58100 Giannitsa, Greece; 5 Department of Animal Production, Faculty of Agriculture, Aristotle University of Thessaloniki, 54124 Thessaloniki, Greece

**Keywords:** olive, olive leaves, olive leaf extract, poultry, broilers, antioxidants, meat quality

## Abstract

Olive leaves are byproducts οf the agro-industrial sector and are rich in bioactive compounds with antioxidant properties. They could be supplemented in poultry diets powdered or less frequently as extracts to improve performance, health and product quality. The objective of this study was to investigate the possible beneficial effects of an aqueous isopropanol olive leaf extract—purified through filtration (250–25 µm) and a resin (XAD-4)—when supplemented in broiler chickens’ diets, on meat quality parameters, focusing mainly on antioxidant parameters as there is limited published information. For this purpose, four-hundred-and-eighty-day-old broilers were randomly assigned to four dietary treatments: T1 (control: basal diet); T2 (1% olive leaf extract); T3 (2.5% olive leaf extract); T4 (positive control: 0.1% encapsulated oregano oil commercially used as feed additive). At the end of the experimental period (day 42), the birds were slaughtered, and samples from breast, thigh meat and liver were collected for antioxidant parameters evaluation. On day 1, after slaughter, in thigh meat, Malondialdehyde (MDA) was lower in T2 compared to T3, and total phenolic content (TPC) was higher in T2 compared to T3 and T4. Total antioxidant capacity (TAC) was increased in T2 and T4 breast meat compared to the control. In liver, T4 treatment resulted in higher TPC. The lack of dose-dependent effect for olive leaf extract may be attributed to the pro-oxidant effects of some bioactive compounds found in olive leaves, such as oleuropein, when supplemented at higher levels. In summary, it can be inferred that the inclusion of 1% olive leaf extract in the feed of broilers has the potential to mitigate oxidation in broiler meat and maybe enhance its quality.

## 1. Introduction

The use of natural, plant-derived feed additives in poultry production has increased in recent years due to restrictions on antibiotic use and consumers’ demand for safe animal products. Many materials of plant origin contain bioactive compounds that offer antioxidant, antimicrobial, immune-protective, antifungal, antiviral, antiparasitic, antitoxigenic and other beneficial properties [1,2,3,4,5]. Moreover, there are natural growth promoters derived from plants that enhance growth performance and have successfully replaced the growth-promoting antibiotics that were banned many years ago [1,2,3]. Among the sources of plant origin that are investigated for applications in the field of feed additives, there is a significant interest in the exploitation of non-conventional sources such as the byproducts and wastes from the agro-industrial sector. Upon the activities of such sector, a significant amount of byproducts is produced, which are disposed of and consist of environmental burden, although they are rich in bioactive compounds. Thus, the practice of using such byproducts as feed additives for livestock is of great importance for the circular economy and environmental protection. However, the appropriate inclusion levels are still under investigation, as they often contain compounds with antinutritional properties. Apart from the inclusion level, the primary issue associated with utilizing byproducts as feed additives is the variation in bioactive compound levels across different batches.

Olive tree (*Olea europaea*), native to the Mediterranean region, is widely cultivated for the production of table olives and olive oil, which are essential components in the Mediterranean diet [6]. In the EU countries, the cultivated area is around 4.6 million ha [7]. As a consequence, large quantities of byproducts are produced with interest for valorization, which is estimated to be around 9.6 million tones/year (olive pomace, olive leaves and stone) and 11.8 million tones/year of biomass deriving from olive tree pruning [8]. In addition to problems caused by the quantity and quality of olive byproducts, there are economical, technical and organizational issues, such as the seasonality of the harvest, which makes a sustainable and environmentally friendly disposal difficult [9]. Olive byproducts contain bioactive compounds; thus, they can be used as feed additives in livestock, and this activity is ranked second together with food application in the pyramid of bioeconomy. In the realm of applied research in this domain, prioritizing olive biomass, such as olive leaves, as a reservoir of bioactive compounds stands as a key οobjective [8]. Olive byproducts provide antioxidant, antimicrobial and anti-inflammatory effects and could also be beneficial for poultry health and performance [10,11].

Olive leaves constitute 10% of the entire olive harvest [12]. Olive leaves and olive leaf extract are used in medicine, cosmetics and as feed additives. They contain bioactive compounds, such as oleuropein, verbascoside, flavonoids, oleanolic acid and mannitol, with potential antimicrobial, antioxidant, anti-inflammatory, antihypertensive, hypocholesterolemic and other beneficial properties [13,14,15,16]. Among these phytochemicals, oleuropein is the main compound responsible for the powerful antioxidant and antimicrobial activity of olive leaf extract [17]. Previous studies in broilers showed that dietary supplementation with olive leaves or their extract improved performance, meat quality, oxidative stress parameters and intestinal health [18,19,20,21,22]. Contrary to these findings, there were reported cases where broilers’ growth performance was not improved [23,24] or even deteriorated [25] following dietary supplementation of olive leaves or their extract. These negative effects may be due to the use of high levels of materials that are also non-purified [11]. Overall, the type of olive byproducts, the inclusion level and the purification are parameters that must be considered by the researchers [26].

The objective of this study was to evaluate the effects of an olive leaf extract, supplemented in the diets of broilers in a low and a higher dose, on lipid peroxidation and total antioxidant capacity. Moreover, encapsulated oregano oil was used as a positive control treatment, as oregano is one of the most popular herbs used as a phytogenic feed additive in poultry production [2]. Oregano essential oil contains the monoterpene carvacol and other phytochemicals responsible for its antimicrobial, antiparasitic, antioxidant and immunomodulatory properties [2,27]. As a feed additive in poultry, it has been shown to increase performance; improve feed intake, conversion and digestion; reduce disease incidence; and improve meat quality by delaying lipid oxidation [27,28,29].

Over recent years, there has been a trend toward the investigation of using olive leaf extracts in broilers’ diets instead of powdered olive leaves as a means to include bioactive phenols rather than other leaf ingredients. Even so, to our knowledge, the studies providing information on the oxidative status of broiler meat are limited (liver [21], breast muscle [22]). Considering the above and the fact that this issue is important for growers and consumers, the present study aims to add further knowledge. Thus, a novel olive leaf extract was obtained with an environmentally friendly technique, characterized by its bioactive content, and was supplemented in broiler diets. Two levels were used (1.0 and 2.5% *w/w*), with the first one similar to those of other studies using leaf extracts in broilers and the second one 2.5-fold higher. The latter was selected within the concept to provide more available bioactives, namely oleuropein, hypothetically expecting more improvement. The addition of a positive control, such as the oregano group, adds depth and innovation to the study.

## 2. Materials and Methods

### 2.1. Ethical Considerations

The experimental protocol was approved by the Ethical Committee branch of the Research Committee of Aristotle University of Thessaloniki, Greece (decision number 246648/15-10-2021; project number 72623). All welfare considerations outlined in the Good Farming Practice Guidelines were taken into consideration when designing the experiment’s animal phase. (Directive 2010/63/EC; Commission recommendation 2007/526/EC).

### 2.2. Raw Materials, Animals, Diets and Experimental Design

The extract used in this study was obtained from olive leaves collected from organically grown olive trees originating from regions of Laconia in Greece during the 2022–2023 harvest period. Specifically, small branches were collected after the pruning of the trees was finished and then placed in a dry room to remove as much humidity as possible over the course of 4 weeks. After that period, relative humidity was found to be approximately 5%, which was sufficient for pulverization of the olive leaves with a typical laboratory blender. Olive powder was then sieved to particles smaller than 0.71 mm and stored at 8–10 °C prior to extraction. The powder was extracted with isopropanol/waterː 7/3 (*v/v*), at a ratio 1:5 Kg/L, with the aid of ultrasounds and stirring. After purification of the solution with sequential filters (250–25 µm) to remove solids and XAD-4 resin to remove non-active ingredients, the extract was condensed and eventually dried appropriately. The resulting material was analyzed by High-Performance Liquid Chromatography with Diode-Array Detection following COI/T.20/Doc. No 29/Rev.1 2017 elution protocol [30] was found to contain oleuropein 22.84 g/100 g extract, whereas Luteolin-7-O-glucoside, verbascoside and hydroxytyrosol were below 0.8 g/100 g extract. The extract also contained triterpenic acids, namely maslinic and oleanolic acid (1.08 and 4.97 g/100 g extract, respectively). The latter was analyzed isocratically, using as mobile a mixture of 0.5% aqueous formic acid (A) and ACN (B) 15:85, *v/v* at a flow rate of 0.8 mL/min (detection 210 nm). (The extraction preparation and the HPLC-DAD analyses were carried out at the Department of Pharmacognosy and Natural Products Chemistry, Faculty of Pharmacy, University of Athens, Athens, 15771, Greece.)

The oregano essential oil utilized in this study was prepared by the vis-Naturalis company. Oregano oil is rich in phenols, the most important of which is carvacrol. The oregano essential oil utilized by vis-Naturalis consists of carvacrol in the range of 78% to 85%, according to their statement. Microencapsulation of oregano oil was achieved by spray drying, resulting in a white powder. Microencapsulation was applied to protect sensitive components of oregano oil from light and humidity [31].

The study duration was 42 days (6 weeks). At the start of the experiment, the chickens were allocated to 4 dietary treatments with 12 replicates that comprised 10 broilers each. The treatments were as follows: T1—control, no additive; T2—olive leaf extract supplementation at 1.0% of the diet; T3—olive leaf extract supplementation at 2.5% of the diet; T4—oregano oil supplementation at 0.1% of the diet (manufacturer: vis-Naturalis, Gennimata 17 str, Kalamaria, Thessaloniki, Postal Code 55132, Greece). A two-phase diet feeding regime was applied: a starter–grower diet (days 1 to 21 of age) and a finisher diet (days 22 to 42 days of age). Diets were wheat-, corn- and soybean-based (Table 1). The lower inclusion level was in accordance with previous studies in broilers, where positive effects were noted. A higher inclusion level was selected to test if any positive effects of the particular olive leaf extract are dose-dependent, making bioactives (namely oleuropein) more available.

At 42 days of age, one broiler per pen and per treatment was randomly selected and euthanized. Electrical stunning was applied prior to slaughter using a VE Memory stunner (FAF, Saint-Sernin-sur-Rance, France). Samples of breast and thigh meat were selected and stored at −20 °C until further analysis.

### 2.3. Feed, Essential Oil and Olive Leaf Extract Analyses

#### 2.3.1. Characterization of Feed Samples

The laboratory samples from each feed obtained from the main batch (1 tn) were 400 g. The samples were then separated into three homogeneous sub-samples. The sub-samples were measured at least once, and values were averaged. In the case of extraction for phenolic content and antioxidant activity, each sub-sample was extracted once, and the resulting extracts were combined into a representative one for further analysis (see below).

The color was assessed using α MiniScan XE Plus D/8S Color Analyzer Colorimeter Spectrophotometer (Hunterlab, VA, USA) as described by Pyrka et al. (2023) [32]. Measurements were carried out 5 times. ∆E values were calculated accordingly [33].

a_w_ was measured at 25 °C using an Aqualab 3TE water activity meter (Decagon Devices Inc., Pullman, WA, USA). Values were determined thrice.

Moisture was determined with the aid of a moisture analyzer DAB 100–3 (Kern & Sohn, GmbH, Balingen, Germany) set at 103 °C. Monitoring was carried out until constant weight (Commission Regulation (EC) No 152/2009) [34]. Measurements were carried out in triplicate.

Crude fat was determined with the aid of a Soxhlet apparatus [34]. Extraction and measurement were carried out in triplicate.

Fatty-acid content was determined with the aid of a gas chromatography system (TRACE GC 2000 Series, Thermo Quest CE Instruments) coupled to a flame ionization detector (FID) and equipped with an autosampler (TRIPLUS AS Thermo Quest CE Instruments) according to Vasilopoulos et al. (2022) [35]. The identification of FAMEs was achieved via a comparison of the retention times (RT) with those of a standard mixture (AccuStandard, New Haven, USA) of 37 fatty acids analyzed under the same chromatographic conditions. Chromatograms were acquired and processed with the assistance of Chrom Quest 5.0 software (ver. 3.2.1, Thermo Separation Products). Measurements were carried out in triplicate.

Crude protein determination was based on the estimated nitrogen content, according to the Kjeldahl method [34]. Measurements were carried out in triplicate.

Crude ash content was determined gravimetrically after combustion at 550 °C until constant weight [34]. Measurements were carried out in triplicate.

Phenolic content and antioxidant capacity determination: Extraction and the determinations of total phenol content (TPC) by the Folin–Ciocalteu assay total flavonoid content (TFC) by the AlCl_3_ method, DPPH^●^ scavenging and Cupric ion Reducing Antioxidant Capacity (CUPRAC), all measured in triplicate with the aid of a UV-1601 spectrophotometer, were carried out as described by Pyrka et al. (2023) [32]. More specifically, extraction was carried out using 1 g of sample and 50 mL methanol (60 °C, 30 min, ultrasound bath, centrifugation 10,000× *g* for 10 min). For TPC, 0.4 mL of extracts reacted with 0.5 mL Folin–Ciocalteu reagent, followed by the addition of 1.5 mL sodium carbonate 20% *w/v* after 3 min and made up with water to 10 mL (measurement at 750 nm after 1 h). The results were expressed as gallic acid equivalents (mg GAE/g feed) using a standard curve (10–120 µg/10 mL, LOQ: 31 µg GA). For TFL, 1.5 mL and 0.1 mL of an AlCl_3_ (2% AlCl_3_ in 95/5 methanol/acetic acid, *v/v*) was added, and subsequently, 0.9 mL of MeOH/acetic acid mixture (95/5, *v/v*) was added up to 2.5 mL (measurement at 415 nm after 30 min). The results were expressed as quercetin equivalents (µg QUE/g feed) using a standard curve (1–50 µg/2.5 mL, LOQ: 13.2 µg). For DPPH^●^, 0.2 mL of the extract was mixed with 2.8 mL of a 0.1 mM DPPH^●^ (measurement at 516 nm after 30 min). The results were expressed as Trolox equivalents (µmol TE/g feed) using a standard curve (0.01–0.12 µmol /3 mL, LOQ: 0.08 µmol). In the *y*-axis, the % radical scavenging (%RSA) value was first calculated. For CUPRAC, 1 mL of Cu(II), Neocuproine and ammonium acetate buffer (pH 7) solutions were mixed. Then, 0.2 mL of extract was added in addition to 0.9 mL water up to 4.1 mL (measurement at 450 nm after 1 h). The results were expressed as Trolox equivalents (µmol TE/g feed) using a standard curve (0.03–0.24 µmol/4.1 mL, LOQ: 0.11 µmol). In all cases, appropriate blanks were used for correction. LOQ was determined from the constructed standard curves as (10 × SD of the y-intercept)/slope.

#### 2.3.2. Characterization of Essential Oil Content and Composition

The oil was isolated from the encapsulated material by the Clevenger hydrodistillation method (~5%). Analysis, identification and quantification with the aid of an Agilent 6890A GC-MS coupled with an MSD 5973 mass spectrometer (Palo alto, CA, USA) was made according to Plati et al. (2021) [36]. Total phenol content and antioxidant capacity were measured following the same procedures for feed extracts. An essential oil solution (5 mg/mL) in methanol was prepared for such a purpose.

#### 2.3.3. Characterization of Olive Leaf Extract

ɑ_w_, moisture content, total phenol, total flavonoid content and antioxidant capacity were measured following the same procedures for feed extracts.

### 2.4. Meat Quality Analyses

#### 2.4.1. Lipid Oxidation

To assess lipid oxidation in broiler meat samples, thiobarbituric acid-reactive substances (TBARS) method was conducted, according to Ahn et al. (1999), with slight adjustments [37]. Tissue samples weighing 1 g were homogenized with a mixture of 8 mL 5% trichloroacetic acid (TCA) and 5 mL 0.8% Butylated Hydroxytoluene (BHT) dissolved in hexane. Subsequently, the samples underwent centrifugation at 3000 g for 5 min, after which 1.5 mL of the underlying layer was collected. To this, 2.5 mL of 0.8% thiobarbituric acid (TBA) was added, and the mixture was incubated in a water bath at 70 °C for 30 min. The resulting sample’s absorbance was measured at 532 nm using a spectrophotometer. Lipid oxidation was determined as the thiobarbituric acid reactive substances (TBARS) value, expressed as ng MDA/g of tissue. The TBARS analysis method was performed on days 1, 5 and 9 after slaughter (breast, thigh meat) or on day 1 (liver).

#### 2.4.2. Total Antioxidant Capacity (TAC) (Phosphomolybdate Method)

The total antioxidant capacity was assessed following the method described by Prieto et al. (1999) [38], using a phosphomolybdate reagent. Tissue extracts (100 μL) were vortexed in Eppendorf tubes with 1 mL of reagent solution (composed of 0.6 M sulfuric acid, 28 mM sodium phosphate and 4 mM ammonium molybdate) and incubated in a water bath at 95 °C for 90 min. Once the samples reached room temperature, the absorbance of each aqueous solution was measured at 695 nm using a blank solution as a reference. The typical blank solution consisted of 1 mL of reagent solution and an appropriate volume of the same solvent employed in the sample, undergoing incubation under identical conditions as the rest of the samples. In the case of samples with unknown compositions, both lipid-soluble and water-soluble antioxidant capacities were quantified in terms of equivalents of α-tocopherol and ascorbic acid, respectively.

#### 2.4.3. DPPH Determination of Radical Scavenging Activity

To determine the radical scavenging activity in chicken tissue, a 25 μL aliquot of the sample was mixed with 975 μL of 2,2-diphenyl-1-picrylhydrazyl (DPPH) solution (100 µM in MeOH), as described by Vasilopoulos et al. (2022) [35]. The mixture was then incubated in the dark for 30 min, followed by measurement of absorbance at 515 nm using a UV-Vis spectrophotometer. A control was prepared by using a DPPH solution without any sample incorporation. A standard curve was constructed utilizing Troloxand the results were expressed as the percentage of radical scavenging activity (% RSA) for each chicken tissue sample, which was calculated using the formula: (% RSA) = [(Ablank − Asample)/Ablank] × 100.

#### 2.4.4. Protein Carbonyls Determination

The method described by Patsoukis et al. (2004) [39] was utilized to determine protein carbonyls in chicken tissue samples. First, 50 μL of 20% TCA was added to 50 μL of the sample homogenate, which was diluted 1:2 *v/v*. The mixture was then incubated in an ice bath and subsequently centrifuged. The resulting supernatant was discarded, and the pellet was treated with 2,4-dinitrophenylhydrazine (DNPH). After an incubation period at room temperature in darkness, the samples were centrifuged again. The supernatant was once again discarded, and 1 mL of 10% TCA was added to the pellet, followed by vortexing and centrifugation. The supernatant was then discarded, and 1 mL of ethanol–ethyl acetate (1:1 *v/v*) was added, vortexed and centrifuged. Next, the supernatant was removed, and 1 mL of 5 mol/L urea (pH 2.3) was added to the remaining pellet. The mixture was vortexed and incubated at 37 °C for 15 min, followed by centrifugation at 15,000× *g* for 3 min at 4 °C. In this assay, the formation of protein carbonyls was detected by the reaction of protein carbonyls with 2,4-DNPH, resulting in the formation of 2,4-dinitrophenylhydrazone (DNP-hydrazone). The absorbance of DNP-hydrazone was measured at 375 nm. The concentration of protein carbonyls was calculated based on the molar extinction coefficient of DNPH and expressed as nmol/mg of protein.

#### 2.4.5. Total Phenol Content (TPC) Determination

The TPC determination was performed on day 1, 5 and 9 after slaughter (breast, thigh meat) or on day 1 (liver). The TPC of tissues was evaluated following the protocol described by Jang et al. (2008) [40]. A total of 2 g of tissue was homogenized with 6 mL of distilled water. Next, 3.6 mL of dichloromethane was added to the homogenate and mixed using a vortex. The sample was then centrifuged, and the resulting supernatant was collected and measured. To prepare the diluted sample, 1 mL of the supernatant was mixed with 4 mL of distilled water. Subsequently, 1 mL of the diluted sample was combined with 500 μL of Folin–Ciocalteu reagent and 1 mL of 7% Na_2_CO_3_ solution. The mixture was thoroughly mixed using a vortex and incubated at room temperature for 60 min. After the incubation period, the total phenolic content was determined using a spectrophotometer set to measure absorbance at 700 nm. The results were expressed as μg of Gallic acid equivalents (GAE) per g of tissue.

### 2.5. Statistical Analysis

The Statistical Package for the Social Sciences (SPSS) software (SPSS 22.0 Version, Chicago, IL, USA) was used to analyze the data. At *p* < 0.05, statistical significance was considered. The sample representing each pen was regarded as the experimental unit. Results are presented as means with standard deviation. One-way ANOVA of the GLM procedure of SPSS was used to analyze the effects of treatments on the tested variables. Tukey’s test was used for post hoc comparisons between treatments.

## 3. Results

### 3.1. Feed, Essential Oil and Olive Leaf Extract Analyses

#### 3.1.1. Feed Color, a_w_ and Proximate Composition

The results on feed color, a_w_ and proximate composition of the treatments are presented in Table 2.

Regarding the color characteristics, the control samples were like those containing the encapsulated essential oil. Thus, they were slightly brighter than those containing olive leaf extract, less green (a* values) and less yellow (b* values) than those containing the extracts. The slightly darker, greener and more yellow were those containing 2.5% olive leaf. Calculation of ΔE values showed that the two batches of control (T1A and T1B) and those containing the encapsulated oil (T4A and T4B) did not differ and are considered indistinguishable by the human eye (ΔE < 0.7). This could be rather expected considering that the encapsulated oregano oil, although in the form of white powder, was added just at the level of 0.1%. Those containing the olive leaf extracts were expected to be visually different as the ΔE values were higher than 1 compared to controls or the samples containing oregano oil. Particularly, for T2A, the ΔE value was ~2; for T3A, it was ~3.5; for T2B, it was ~2.6; and for T3B, it was ~4.0, respectively. The two batches, A and B, containing the same level of olive leaf extract, also did not differ in terms of color, as the ΔE values were in the range of 0.2 to 0.6. Such findings, considering the green-yellow coloration of the olive leaf extract, are indicative of the adequate mixing of the feed constituents and the different doses of the extract. The a_w_ values of the produced feeds were less than 5.5; thus, except for low enzymatic activity, no growth of bacteria, yeast and mold is expected as they should contain only capillary adsorbed water and/or less strongly bound water layers, but no free one [41]. Additionally, moisture content is below 10%. The corresponding a_w_ and moisture content values were all lower compared to the literature values for different feeds, including a couple appropriate for chicken [42], indicating their high stability. Concerning the other parameters, the variability observed was rather small, and any statistical differences were rather related to sample homogeneity and analytical determinations rather than an effect of the additive, granted that an extract was used and not olive leaves, whereas the level of addition of the essential oil was 10- and 25-fold lower compared to the levels of the extract.

#### 3.1.2. Feed Fatty Acid Profile

The results of fatty acid profile analysis of the diets are presented in Table 3.

The fat content of the feed was rich in polyunsaturated fatty acids, as expected due to the use of soya and corn, and as evident, their level was almost 58%, followed by mono-unsaturated (~25.5%) containing saturated at~15.5%.

#### 3.1.3. Feed Antioxidant Parameters

The antioxidant parameters measured in feed samples (TPC, TFC, DPPH, CUPRAC) are presented in Table 4.

#### 3.1.4. Characterization of Essential Oil Content and Composition

To be able to extract the oil and disrupt the wall material, hydrodistillation was used. The calculated % was ~5% *w*/*v* in accordance with the manufacturer. The GC-MS analysis identified thymol and carvacrol as the main components. The major compounds and their % concentration were as follows: carvacrol (63.91%), thymol (24.90%), caryophyllene oxide (2.68%), bisabolene (2.68%), borneol (1.68%), terpineol (0.79%), trans-dihydrocarvone (0.26%) and caryophyllene (2.75%), generally in accordance with the literature for *Origanum vulgare ssp. hirtum* [43,44]. The absence of *p*-cymene, as well as some discrepancies compared to the declaration of the company, may be related to the effect of the isolation process from the encapsulated material [36]. The TPC and antioxidant capacity values were as follows: TPC (60.2 ± 8.8 mg GAE/g oil, *n* = 3), DPPH^●^ (0.42 ± 0.03 mmol TE/g oil, *n* = 3), CUPRAC (0.68 ± 0.05 mmol TE/g oil, *n* = 3). These values, for the aforementioned reasons, should be considered with caution as the pure original oil was not available for analysis.

#### 3.1.5. Characterization of Olive Leaf Extract

Dry leaf extract ɑ_w_ was 0.258 ± 0.001, and moisture content was <10%. Regarding olive dry leaf extract antioxidant parameters measured, they were as follows: TPC: 157.8 ± 8.8 mg GAE/g extract (*n* = 3); TFL: 16.4 ± 0.1 mg QUE/g extract (*n* = 3); DPPH^●^: 1.02 ± 0.07 mmol TE/g extract (*n* = 3); CUPRAC: 1.54 ± 0.07 mmol TE/g extract (*n* = 3). As evident, the extract was dry, containing a strongly bound water monolayer, with a moisture content of <10%, as required by Pharmacopoeia [45]. The phenol content and antioxidant activity values measured justify the findings in the prepared feeds. The almost equal activity of the control with those of the samples containing the essential oil should be related to the 0.1% addition (5- and 10-fold lower than the olive leaf extract), as well as to the encapsulated form of the oil used, which offers protection to active constituents.

### 3.2. Meat Quality Analyses

#### 3.2.1. Lipid Oxidation

Differences in MDA content were noticed only in the thigh samples measured on the 1st day of collection, where the values were lower by average in T2 and T4 in comparison with T3 (*p* = 0.026). Although not statistically significant, it can be noticed that T2 meat samples had consistently lower MDA levels compared to the other groups in all three days of analyses (day 1, 5 and 9). In liver samples examined on the first day, no statistically significant differences were found, with those of T4 and T2 samples being the lowest. The results are presented in Table 5.

#### 3.2.2. Total Antioxidant Capacity (TAC)

Total antioxidant capacity (TAC) was elevated in T2 and T4 groups in breast meat, as compared to T1 (*p* = 0.050). In the other tissues, no differences were noted among groups. The results are presented in Table 6.

#### 3.2.3. DPPH-RSA Determination

No significant differences were found for DPPH radical scavenging activity (%RSA) among the treatments. The results are presented in Table 7.

#### 3.2.4. Protein Carbonyls Determination

No significant differences were found for protein carbonyls among the treatments. The results are presented in Table 8.

#### 3.2.5. Total Phenol Content Determination

The results obtained from TPC analysis are presented in Table 9. TPC was higher in thigh meat, day 1, in the T2 group in comparison with T3 (*p* = 0.044), but no differences among groups were noticed for days 5 and 9. Regarding the TPC of breast meat, there were no differences among the groups. Liver TPC was significantly increased in T4 treatment (*p* < 0.001).

## 4. Discussion

Based on the existing literature regarding the high nutritional value of olive byproducts and their beneficial effects reported in poultry, this study was designed to investigate how dietary olive leaf extract at two levels can affect meat quality in broilers. A previous study on broilers showed that their supplementation with olive leaf extract in drinking water improved the performance of birds [18]. In another study, the dietary supplementation of olive leaves in broilers improved meat quality, as it lowered protein and lipid oxidation, delayed the deterioration of flavor and odor, increased juiciness and reduced acidity [19]. Similarly, supplementing 0.3% olive leaf extract in broilers improved breast meat antioxidation and positively affected caecum microflora [22]. In the study of da Silva et al. (2018) [20], dietary supplementation of 0.5% and 1% olive leaves in Cobb chickens reduced primary lipid oxidation products in processed meat products. Dietary olive leaf extract supplementation as an ethanol solution (5 mL/kg diet containing 53.0 mg oleuropein/mL) to broilers, although having some positive effects in parameters of oxidative stress measured in blood and intestinal health, did not show any effect on liver lipid oxidation as estimated by MDA measurement [21]. In the present study, the levels of olive leaf extract were selected based on the findings of the previous studies. A second level was used 2-fold higher to examine whether a stronger effect would be observed via making the extract bioactives (namely oleuropein) available at a higher level. Oregano essential oil, a plant oil rich in phytochemicals and commonly used in poultry as a feed additive, was used as a positive control in T4 treatment to investigate if it excels in any of the parameters studied.

In all MDA measurements, T2 treatment reduced numerically the MDA values of chicken breast, thighs and liver in comparison with the control. There was also a significant difference in MDA levels between T2 and T3 thigh meat on day 1, as already described. These results indicate that olive leaf extract supplementation at the lower level (1%) caused retardation of lipid oxidation in tissues, as MDA is considered the main product for the evaluation of lipid peroxidation [46]. Lipid peroxidation is one of the most important procedures that negatively affect the quality and shelf-life of animal products [47]. It causes adverse effects on the organoleptic properties and nutritional value of meat and meat products [47]. Olive byproducts can be used as natural antioxidants because of their high content of phenolic substances [48]. In particular, olive leaf extract contains phenols (oleuropein, verbascoside) and flavonoids responsible for their antioxidant properties [21,22], which explain the beneficial effects on lipid and protein peroxidation found in this study. Numerous previous studies have reported similar effects on MDA values following dietary supplementation of olive byproducts on poultry meat [19,22,48,49] and liver [50,51]. Moreover, T4 thigh meat had similar MDA levels on day 1 of analysis as T2, indicating similar effects on lipid oxidation. This finding is in line with previous studies in broilers and turkeys, which showed that dietary oregano supplementation provides beneficial effects on the lipid oxidation of meat [52,53,54,55]. It can be noticed that the higher inclusion level of olive leaf extract did not exhibit beneficial effects on lipid oxidation, potentially due to the higher concentration of components that could act as pro-oxidants [56].

Similar results were found for TAC in breast samples, with T2 and T4 groups having higher TAC levels. The phosphomolybdate assay was used to assess TAC in tissue extracts. In general, the oxidative stability of meat depends on the content of antioxidants, pro-oxidants and substrates prone to oxidation, such as the polyunsaturation degree of fatty acids [57,58]. Chicken meat, due to its high degree of lipid unsaturation, may be prone to oxidative damage [58]. The findings of our study indicate that the antioxidant compounds found in olive leaf extract and oregano essential oil (positive control) can enhance the antioxidant capacity in breast meat. However, higher doses of olive leaf extract (T3 group) did not have the same effects on TAC in breast meat. Regarding TAC in thigh meat, it was even numerically lower in the T3 group compared to the control. The lack of consistent effects of the higher olive leaf inclusion level (2.5%) may be due to imbalances in the amounts of antioxidants, pro-oxidants and substrates in the meat, as discussed above. However, previous studies in poultry showed that TAC in plasma was increased following dietary supplementation of olive leaf extract [59] or mill wastewater by products [60]; no similar responses have been recorded for tissues so far.

The DPPH-RSA assay is a widely used method based on a mixed-mode electron/hydrogen transfer for the determination of antioxidant activity. Based on the existing literature about the high polyphenol content of olive byproducts, it was assumed that molecules that could act as radical scavengers, such as oleuropein found in olive leaves, could inhibit oxidation [48]. However, no significant differences were found among treatments in any of the examined tissues. Our results agree with a previous study [61], where DPPH analysis was performed in fresh or processed thigh meat samples obtained from broilers treated with a freeze-dried powder from organic olive and showed no effect of the dietary supplement on DPPH-RSA values. In contrast, in another study, the DPPH assay showed higher antioxidant activity in meat from broilers supplemented with a semi-solid olive cake [48]. These inconsistencies may be due to the different olive byproducts, the different phenolic compounds and the different doses used in these studies.

Protein carbonyls are biomarkers for the evaluation of protein oxidation [62]. Protein oxidation is a procedure that deteriorates meat quality by inducing changes in muscle proteins, such as denaturation and proteolysis [49]. These changes can negatively affect meat color, aroma, flavor, texture parameters and water-holding capacity. Although there were no statistically significant differences among treatments in our study, protein carbonyls tended to reduce in T2 breast samples (*p* = 0.053). Similar effects on breast meat protein oxidation have been previously reported in studies where different olive byproducts were supplemented in broilers (olive mill wastewater and dried olive pulp) [49,51]; however, to our knowledge, this is the first study that used olive leaf extract and reported this tendency. According to Tufarelli et al. (2022) [51], high levels of dried olive pulp may result in high auto-oxidation rates, negatively affecting the equilibrium between pro- and antioxidative procedures in meat. Although the byproduct was different, a similar mechanism is possibly involved in our study. As stated by Schicchitano et al. (2023) [56], oleuropein, as well as most of the phytochemicals present in a Mediterranean diet, can both act in biological systems as antioxidants and pro-oxidants. The latter is expected to be dose-dependent, and as stressed by the authors, it can be beneficial at low levels and present negative effects at higher ones. Shimao et al. [63] showed that supplementing pure oleuropein in growing broiler diets at too low levels (0.1, 0.5 and 2.5 mg/kg) resulted in significant antioxidant protection in breast oxidation in terms of carbonyls for levels of 0.5 and 2.5 mg/kg compared to control. However, no difference in protein carbonyls was observed between these two levels, and the authors did not introduce a much higher level in the diet to show a negative effect. Based on their overall findings, they suggested that administration of oleuropein may result in an effect in the ‘mitohermetic’ pathway involving transcriptional, etc., changes and can be harmful when ingested at high levels. Another suggestion from a past study is that under specific conditions and at high concentrations, oleuropein and hydroxytyrosol can act as pro-oxidants by producing H_2_O_2_ in significant amounts [64]. Furthermore, as shown in vitro by Mazziotti et al. [65], it can recycle ferric ions, which can induce the decomposition of hydrogen peroxide to harmful hydroxy radicals following a Fenton-type reaction.

Olive byproducts contain high concentrations of phenolic compounds, which are bioactive metabolites derived from plant species [66]. It was assumed that the phenolic compounds found in olive leaf extract could be transferred to the tissues of broiler chickens and enrich the nutritional value of meat. Our results showed that olive leaf extract supplementation did not increase the TPC of meat compared to a basal diet (control). However, T2 treatment resulted in higher TPC in the thigh on day 1 compared to the higher olive leaf extract dose (T3) or the positive control (T4), indicating lower oxidation of the phenolic compounds. This finding is in accordance with the effects of T2 treatment on other antioxidant parameters (MDA, TAC) in meat, which have already been described previously. At present, there is no clear evidence, but it could be possible considering the findings of a study with a very different byproduct (olive cake) containing related compounds to olive leaf, where traces of tyrosol and metabolites of hydroxytyrosol were detected in chicken meat [49]. Moreover, the positive control treatment (T4) increased TPC in the liver on day 1. Even though the literature investigating how oregano essential oil affects liver phenolic content is limited, it has been shown that supplementing an extract with 5% oregano and 0.5% sage essential oils in chicken diet can increase the TPC of breast and thigh meat [67]. Similarly, oregano essential oil alone or combined with laurel essential oil, when supplemented in broilers’ diet, increased TPC in breast and thigh meat [68]. Further investigation is warranted to better understand phenol bioavailability and tissue deposition in chicken. Even if not accumulated in tissues, phenolic metabolites might also exert local antioxidant activity due to their prolonged circulation in the body [69]. Before concluding, it should be highlighted that in the in vivo systems, the bioavailability of phenols is a very important factor, as transformations of molecules may occur into derivatives with different properties [70]. Oleuropein and related compounds present structural characteristics (catechol moiety, ester and glucosidic bonds) that make them prone to changes during the different phases of metabolism. Eventually, hydroxytyrosol and elenolic acid are expected to form, and these are converted to sulfate derivatives. Such a derivative, e.g., for hydroxytyrosol using theoretical calculations, was predicted to be rather inactive as a radical scavenger [71]. Hydroxytyrosol is also reported to be converted to homovanilic derivatives through methylation [70]. The scarce studies, i.e., those of Branciari et al. (2017) [48], who found minute or traces of olive-related phenols in chicken breast meat, may be related to the fact that they did not search for other possible metabolites. According to the review of Nikou et al. (2022) [70], derivatives of hydroxytyrosol, e.g., are distributed in animal tissues. Among these tissues, one where they accumulate the most is the liver. On the other hand, in the liver, oxidation, hydrolysis and reduction may occur, including conjugation. The latter could be partially avoided when encapsulation has been used [72], as in the case of oregano essential oil, which may justify some of the above findings. In addition, the active phenols, such as thymol and carvacrol, are much more lipophilic and of lower molecular weight, suggesting that they should be easier to absorb and, thus, be more bioavailable [70]. The low level used may not necessarily be an issue, considering the findings of Shimao et al. [63] regarding the efficiency of low levels of oleuropein.

## 5. Conclusions

Under the tested circumstances and based on the findings of the study, it can be concluded that 1% dietary aqueous isopropanol olive leaf extract purified through filtration and XAD-4 resin supplementation in broilers can result in lower MDA and higher TPC levels in thigh meat compared to the higher inclusion level (2.5%). In breast meat, both 1% olive leaf oil and 0.1% encapsulated oregano essential oil treatments increased TAC compared to a basal diet. Oregano essential oil, representing the positive control treatment, excelled in the TPC of liver. Overall, it can be concluded that 1% olive leaf extract in broilers’ diet can reduce the oxidation procedures in meat and, therefore, improve meat oxidative status.

## Figures and Tables

**Table 1:** Main ingredients and nutrient analysis of the diets.

Ingredients (%)	Starter–Grower Diet (Days 1–21)	Finisher Diet (Days 22–42)
Wheat soft	50.4	61.8
Corn	15.0	10.0
Soybean meal (47% crude protein content)	28.5	22.7
Soybean oil	2.15	2.85
Limestone	1.30	0.90
Monocalcium phosphate	0.15	0.18
Sodium chloride	0.15	0.18
Sodium bicarbonate	0.28	0.23
Lysine-HCl	0.36	0.22
DL-Methionine	0.27	0.19
DL-Threonine	0.12	0.06
Vitamins and minerals	1.32	0.69
Calculated analysis (%)		
Crude protein	21.5	19.5
Crude fiber	2.51	2.44
Crude fat	3.79	4.44
Crude ash	5.10	4.01
Metabolizable Energy (kcal/kg of diet)	3050	3150

**Table 2:** Color (L*, a*, b* values), a_w_ and proximate composition of feed samples.

Sample Code	L*	a*	b*	Water Activity (a_w_)	% Moisture	% Crude Fat	% Crude Protein	% Crude Ash
T1A	43.4 ± 0.1	3.2 ± 0.1	13.7 ± 0.3	0.531 ± 0.001	9.37 ± 0.16	3.8 ± 0.2	20.1 ± 0.2	4.9 ± 0.1
T1B	43.9 ± 0.1	3.3 ± 0.1	13.6 ± 0.2	0.518 ± 0.003	9.06 ± 0.68	4.4 ± 0.4	18.9 ± 0.3	4.5 ± 0.0
T2A	41.8 ± 0.2	3.1 ± 0.1	14.8 ± 0.3	0.542 ± 0.001	9.20 ± 0.18	3.8 ± 0.1	20.9 ± 0.1	5.1 ± 0.0
T2B	41.7 ± 0.3	3.0 ± 0.1	15.0 ± 0.2	0.542 ± 0.004	8.73 ± 0.54	4.1 ± 0.2	19.1 ± 0.1	4.6 ± 0.0
T3A	41.5 ± 0.2	2.3 ± 0.1	16.2 ± 0.2	0.538 ± 0.002	9.63 ± 0.51	3.5 ± 0.2	20.6 ± 0.3	5.2 ± 0.0
T3B	41.0 ± 0.1	2.5 ± 0.1	16.2 ± 0.4	0.523 ± 0.003	8.51 ± 1.03	4.2 ± 0.2	18.6 ± 0.2	4.6 ± 0.1
T4A	42.9 ± 0.3	3.3 ± 0.2	14.2 ± 0.2	0.495 ± 0.008	9.42 ± 0.27	3.7 ± 0.1	20.4 ± 0.3	5.5 ± 0.6
T4B	43.0 ± 0.1	3.5 ± 0.1	13.6 ± 0.2	0.556 ± 0.001	9.08 ± 0.16	4.2 ± 0.2	18.9 ± 0.4	4.7 ± 0.2

T1: control; T2: basal diet with 1% olive leaf extract; T3: basal diet with 2.5% olive leaf extract; T4: basal diet with 0.1% encapsulated oregano oil. Values are means ± SD (*n* = 5 for color parameters and 3 for others). The letters A and B represent two different feed samples obtained from the same group.

**Table 3:** Fatty acid composition (%) of crude fat contained in feed samples.

Sample Code	16:0	18:0	18:1 (*n*–9)	18:1 (*n*–7)	18:2	18:3 (*n*–6)	20:1	18:3 (*n*–3)
T1A	11.4 ± 0.2	3.5 ± 0.2	23.8 ± 0.7	1.5 ± 0.1	52.3 ± 0.6	5.2 ± 0.7	0.6 ± 0.0	0.6 ± 0.0
T1B	13.1 ± 0.1	3.4 ± 0.1	24.1 ± 0.4	1.7 ± 0.0	50.5 ± 0.7	4.8 ± 0.1	0.8 ± 0.2	0.7 ± 0.1
T2A	10.7 ± 0.2	3.3 ± 0.1	23.5 ± 0.2	1.5 ± 0.1	52.5 ± 0.4	5.4 ± 0.2	0.6 ± 0.1	0.9 ± 0.0
T2B	10.4 ± 0.4	3.6 ± 0.1	23.5 ± 0.2	1.6 ± 0.0	52.5 ± 0.6	5.5 ± 0.1	0.6 ± 0.0	0.9 ± 0.0
T3A	12.1 ± 0.2	3.7 ± 0.2	23.3 ± 0.7	1.9 ± 0.1	50.5 ± 0.6	5.9 ± 0.7	0.9 ± 0.0	0.6 ± 0.0
T3B	11.9 ± 0.2	3.6 ± 0.1	23.1 ± 0.2	1.6 ± 0.1	51.5 ± 0.4	5.2 ± 0.2	0.8 ± 0.1	0.5 ± 0.0
T4A	10.9 ± 0.2	3.3 ± 0.1	24.0 ± 0.0	1.5 ± 0.1	52.4 ± 0.1	5.1 ± 0.1	0.7 ± 0.1	0.8 ± 0.2
T4B	9.5 ± 0.4	3.4 ± 0.1	24.0 ± 0.2	1.5 ± 0.0	52.7 ± 0.6	5.6 ± 0.1	0.7 ± 0.0	0.7 ± 0.0

T1: control; T2: basal diet with 1% olive leaf extract; T3: basal diet with 2.5% olive leaf extract; T4: basal diet with 0.1% encapsulated oregano oil. Values are means ± SD *(n* = 3). The letters A and B represent two different feed samples obtained from the same group.

**Table 4:** Total phenol content (TPC), total flavonoid content (TFL), DPPH scavenging (DPPH) and cupric ion reducing antioxidant capacity (CUPRAC) of feed.

	TPC	TFL	DPPH	CUPRAC
Sample Code	mg GAE/g Feed	μg QUE/g Feed	μmol TE/g Feed	
T1A	1.08 ± 0.02 ^a^	40.8 ± 0.8 ^a^	2.2 ± 0.2 ^a^	13.7 ± 2.0 ^a^
T1B	1.17 ± 0.02 ^b^	32.6 ± 1.3 ^b^	1.4 ± 0.4 ^b^	14.6 ± 0.6 ^a^
T2A	2.91 ± 0.06 ^c^	177.5 ± 6.0 ^c^	12.1 ± 1.0 ^c^	28.3 ± 3.0 ^b^
T2B	2.70 ± 0.07 ^d^	174.5 ± 2.3 ^c^	11.7 ± 1.0 ^c^	33.1 ± 6.4 ^b^
T3A	4.55 ± 0.15 ^e^	375.4 ± 7.2 ^d^	25.5 ± 2.0 ^d^	50.9 ± 2.7 ^c^
T3B	4.37 ± 0.27 ^e^	336.8 ± 1.5 ^e^	25.4 ± 1.9 ^d^	48.5 ± 3.9 ^c^
T4A	1.27 ± 0.07 ^f^	46.9 ± 1.3 ^f^	2.7 ± 0.2 ^e^	12.7 ± 2.4 ^a^
T4B	1.13 ± 0.11 ^b^	33.4 ± 0.8 ^b^	1.8 ± 0.3 ^a^	12.9 ± 0.8 ^a^

T1: control; T2: basal diet with 1% olive leaf extract; T3: basal diet with 2.5% olive leaf extract; T4: basal diet with 0.1% encapsulated oregano oil. Values are means ± SD (*n* = 3). Values in the same column with different superscripts differ significantly (*p* ≤ 0.05). The letters A and B represent two different feed samples obtained from the same group.

**Table 5:** MDA content (ng MDA/g tissue) in broilers’ breast, thigh and liver, evaluated on day 1, 5 and 9 after slaughter (breast, thigh) or on day 1 (liver).

	Treatments				*p*-Value
	T1	T2	T3	T4	
Breast MDA content (ng MDA/g)					
Day 1	1.25 ± 1.130	0.72 ± 0.471	0.89 ± 0.525	2.07 ± 2.436	0.125
Day 5	2.45 ± 1.907	1.74 ± 2.080	4.23 ± 4.854	2.61 ± 2.160	0.237
Day 9	9.97 ± 6.325	3.58 ± 2.567	9.67 ± 7.761	6.57 ± 2.335	0.074
Thigh MDA content (ng MDA/g)					
Day 1	4.57 ± 4.116 ^ab^	2.33 ± 2.547 ^b^	6.03 ± 4.169 ^a^	2.34 ± 2.368 ^b^	0.026
Day 5	5.61 ± 4.867	2.06 ± 2.304	7.04 ± 7.853	6.87 ± 3.927	0.079
Day 9	30.87 ± 24.686	20.25 ± 13.643	35.36 ± 26.354	34.64 ± 21.820	0.558
Liver MDA content (ng MDA/g)					
Day 1	15.87 ± 11.448	9.70 ± 4.565	11.56 ± 3.707	8.75 ± 4.252	0.064

T1: control; T2: basal diet with 1% olive leaf extract; T3: basal diet with 2.5% olive leaf extract; T4: basal diet with 0.1% encapsulated oregano oil. Values are means ± SD (*n* = 12). Values in the same row with different superscripts differ significantly (*p* ≤ 0.05).

**Table 6:** Total antioxidant capacity (TAC) (%) in broilers’ thigh, breast and liver.

	Treatments				*p*-Value
	T1	T2	T3	T4	
TAC (%)					
Thigh	18.8 ± 8.41	21.0 ± 9.20	14.3 ± 5.78	19.6 ± 19.58	0.412
Breast	28.2 ± 16.03 ^b^	46.0 ± 16.62 ^a^	42.8 ± 9.09 ^ab^	50.6 ± 20.01 ^a^	0.050
Liver	54.3 ± 8.05	66.5 ± 16.07	58.3 ± 9.21	53.0 ± 10.16	0.099

T1: control; T2: basal diet with 1% olive leaf extract; T3: basal diet with 2.5% olive leaf extract; T4: basal diet with 0.1% encapsulated oregano oil. Values are means ± SD (*n* = 8). Values in the same row with different superscripts differ significantly (*p* ≤ 0.05).

**Table 7:** DPPH-RSA (%) in broilers’ thigh, breast and liver.

	Treatments				*p*-Value
	T1	T2	T3	T4	
DPPH-RSA (%)					
Thigh	27.3 ± 7.61	25.0 ± 6.73	28.4 ± 5.12	25.5 ± 9.51	0.842
Breast	37.0 ± 5.52	38.0 ± 5.56	37.6 ± 9.15	38.2 ± 4.13	0.989
Liver	56.1 ± 6.23	56.7 ± 9.29	50.8 ± 7.34	57.1 ± 9.35	0.516

T1: control; T2: basal diet with 1% olive leaf extract; T3: basal diet with 2.5% olive leaf extract; T4: basal diet with 0.1% encapsulated oregano oil. Values are means ± SD (*n* = 6).

**Table 8:** Protein carbonyls (nmol/mg of protein) in broilers’ liver, thigh and breast. T1: control; T2: basal diet with 1% olive leaf extract; T3: basal diet with 2.5% olive leaf extract; T4: basal diet with 0.1% encapsulated oregano oil. Values are means ± SD (*n* = 4).

	Treatments				*p*-Value
	T1	T2	T3	T4	
Protein carbonyls (nmol/mg of protein)					
Liver	10.9 ± 4.75	8.6 ± 2.73	10.2 ± 5.77	9.8 ± 3.66	0.901
Thigh	6.6 ± 1.36	6.4 ± 1.66	6.6 ± 2.02	7.5 ± 3.51	0.899
Breast	11.1 ± 3.00	6.4 ± 1.96	9.3 ± 1.14	11.1 ± 3.09	0.053

**Table 9:** Total phenol content (TPC, μg GAE/g tissue) in broilers’ breast, thigh and liver, evaluated on day 1, 5 and 9 (breast, thigh) or on day 1 (liver) after slaughter.

	Treatments				*p*-Value
	T1	T2	T3	T4	
Breast TPC (μg GAE/g)					
Day 1	1227.8 ± 521.71	1236.4 ± 649.34	1168.4 ± 516.06	1388.2 ± 605.22	0.814
Day 5	1581.2 ± 442.46	1673.2 ± 566.74	1404.7 ± 415.79	1681.4 ± 451.34	0.455
Day 9	1653.6 ± 448.58	1710.3 ± 569.63	1449.6 ± 426.42	1707.8 ± 450.96	0.500
Thigh TPC (μg GAE/g)					
Day 1	1463.6 ± 555.89 ^ab^	1621.3 ± 471.84 ^a^	1208.3 ± 306.52 ^b^	1196.0 ± 235.47 ^b^	0.044
Day 5	1654.2 ± 583.37	1872.4 ± 363.47	1629.1 ± 605.02	1843.1 ± 359.35	0.509
Day 9	1742.6 ± 599.5	1916.2 ± 359.38	1717.9 ± 678.85	1657.2 ± 516.28	0.693
Liver TPC (μg GAE/g)					
Day 1	1332.2 ± 1021.20 ^b^	1596.5 ± 1044.30 ^b^	1588.9 ± 1070.77 ^b^	4882.0 ± 979.00 ^a^	<0.001

T1: control; T2: basal diet with 1% olive leaf extract; T3: basal diet with 2.5% olive leaf extract; T4: basal diet with 0.1% encapsulated oregano oil. Values are means ± SD (*n* = 12). Values in the same row with different superscripts differ significantly (*p* ≤ 0.05).

## Data Availability

Data are available upon request.
